# Comparison of air temperature measured in a vineyard canopy and at a standard weather station

**DOI:** 10.1371/journal.pone.0234436

**Published:** 2020-06-11

**Authors:** Andrés Javier Peña Quiñones, Gerrit Hoogenboom, Melba Ruth Salazar Gutiérrez, Claudio Stöckle, Markus Keller

**Affiliations:** 1 AgWeatherNet Program, Washington State University, Prosser, Washington, United States of America; 2 Department of Biological Systems Engineering, Washington State University, Pullman, Washington, United States of America; 3 Department of Horticulture, Irrigated Agriculture Research and Extension Center, Washington State University, Prosser, Washington, United States of America; George Mason University, UNITED STATES

## Abstract

The complex environment within a crop canopy leads to a high variability of the air temperature within the canopy, and, therefore, air temperature measured at a weather station (WS) does not represent the internal energy within a crop. The objectives of this study were to quantify the difference between the air temperature measured at a standard WS and the air temperature within a six-year-old vineyard (cv. Chardonnay) and to determine the degree of uncertainty associated with the assumption that there is no difference between the two temperatures when air temperature is used as input in grapevine models. Thermistors and thermocouples were installed within the vine canopy at heights of 0.5 m and 1.2 m above the soil surface and immediately adjacent to the berry clusters. In the middle of the clusters sensors were installed to determine the temperature of the air surrounding the clusters facing east and west. The data were recorded within the canopy from December 2015 to June 2017 as well as at the standard WS that was installed close to the vineyard (410 m). Significant differences were found between the air temperatures measured at the WS and those within the vineyard during the summer when the average daily minimum air temperature within the canopy was 1.2°C less than at the WS and the average daily maximum air temperature in the canopy was 2.0°C higher than at the WS. The mean maximum air temperature measured in the clusters facing east was 1.5°C higher and west 4.0°C higher than the temperature measured at the WS. Therefore, models that assume that air temperature measured at a weather station is similar to air temperature measured in the vineyard canopy could have greater uncertainty than models that consider the temperature within the canopy.

## 1. Introduction

In general, there is a significant effect of air temperature on growth and development of plants, insects, and diseases, ultimately impacting final crop yield [1; 2; 3; 4; 5]. For grapevine, several studies have been conducted to determine the temperature response for different processes, especially the temperature range where growth and development are optimal [6; 7; 8; 9]. The results from the studies by the aforementioned authors point to air temperature data as a key input for decision making in agriculture in general and especially for viticulture, where temperature not only affects yield quantity but also yield quality through the composition of the grapes [10; 11; 12]. Although the use of air temperature from standard weather stations is a widespread practice in agriculture, temperature readings do not reflect the internal energy of the canopy.

Air temperature at an automated weather station is normally measured above the soil surface under standard conditions following the World Meteorological Organization protocol [13]. These standard installation practices guarantee an unbiased comparison of temperatures among various locations, but they do not make the data representative of the temperature of the air inside or surrounding a crop canopy. Within a crop canopy, the complex environment between the soil surface and the canopy, including the air spaces and leaves, generates high air temperature variability as a response to different patterns of energy exchange [14; 15; 16]. Based on the principles of the energy balance and empirical evidence, several studies have emphasized the need for site-specific weather data to correctly represent the environment inside the crop canopy [17; 18; 19; 2]. Despite studies that have shown the benefit of using on-site air temperature measurements [2; 20], specifically under irrigated conditions, the difference between the two temperatures is still largely unknown. The results reported by Atkinson and Porter [2] and Fatnassi et al. [20] suggested that agricultural models, especially those used to predict the impact of pests on crop yield, performed better by using canopy air temperature. These results could be related to the fact that several models, especially those that use weather variables for prediction of growth and development, are mostly calibrated under controlled environments.

During the past 20 years, several models have been developed for estimating the temperature within a canopy [21; 22; 15]. These models are an effective option to solve the problems associated with the acquisition of site-specific weather data without losing the benefits of on-site information [3]. Matese et al. [23] studied the atmospheric variables in grapevines and reported some differences between the air temperature measured inside the grapevine canopy and outside the vineyard. They found differences between 0.6 and 1.5°C, which were attributed to the type of pruning method. Schultz [6] found that the geometrical structure of the grapevine canopy and its density can affect the energy fluxes at the canopy scale. Because of the impact of air temperature on grape quality, differences between air temperature within the canopy and the berry temperature or berry skin temperature, which can be as high as 12 °C, also have been studied. The results showed that berry skin temperature is always higher than the ambient air temperature even for shaded berries [24; 12]. Nevertheless, only a few studies have examined the differences between air temperature measured at a standard weather station near the vineyard and the air temperature within a grapevine canopy. The aim of this study was twofold. The first objective was to determine differences between the temperature of the air surrounding the canopy vines in a vineyard and the air temperature measured under standard conditions at an automated weather station. Significant differences between air temperature and canopy temperature would mean another source of uncertainty in addition to model parameters and model structure [25; 26]. It is important to note that this study did not consider a fair comparison, i.e., the temperature measured at a height of 1.2 m at the weather station and at a height of 2 m within the vineyard. Instead, we were interested in a comparison of an actual scenario for a decision-makers that is based on weather data measured at the weather station for determining the management of a vineyard with its own micro-climate. The second objective was to examine the degree to which these two temperatures are related to define the degree of uncertainty associated with the use of air temperature data measured at a weather station as input for a grapevine model.

## 2. Methods

### 2.1 Location and general information

This study used the air temperature data measured in the canopy of a vineyard and air temperature measured by a standard automatic weather station. The vineyard and the weather station were both located on the Roza Farm of the Irrigated Agriculture Research and Extension Center (IAREC) of Washington State University in Prosser, Washington (46.29°N, 119.73°W, 359 masl). The Roza Farm is based in the Yakima Valley, a premier growing region for irrigated tree fruit crops, grapes, and alfalfa. The grape vines were an own-rooted *Vitis vinifera* L. cv. Chardonnay. The vineyard was planted in 2010 at a density of 2,035 vines per hectare, with 2.7 m between north-south-oriented rows and 1.8 m within rows. The vines were trained to a bilateral cordon, spur-pruned with shoots loosely positioned between two pairs of foliage wires, and drip-irrigated using regulated deficit irrigation. The weather station was sited at a mean distance of 415 m from the vineyard, a distance considered within the radius of influence of an automated weather station [27].

### 2.2 Air temperature sensors

#### a. Measurements at the weather station

Two air temperature sensors were installed at a height of 1.7 m in open terrain over a grass surface following the standard conditions recommended by WMO [13]. The temperature was measured with two “class A” sensors installed at a height of 1.7 m in naturally aspirated multiplate radiation shields (RM Young Company, Travers City, Michigan, USA). The first sensor was a CS-107 probe (Campbell Scientific, Logan, Utah, USA), which has a thermistor encapsulated in an epoxy-filled aluminum housing and measures air temperature from -35° to +50°C. The second sensor was a HC2-S3 probe (Rotronic, Hauppauge, New York, USA) which measures relative humidity and air temperature from -50 to +80°C.

#### b. Measurements within the canopy

**Fig 1:**
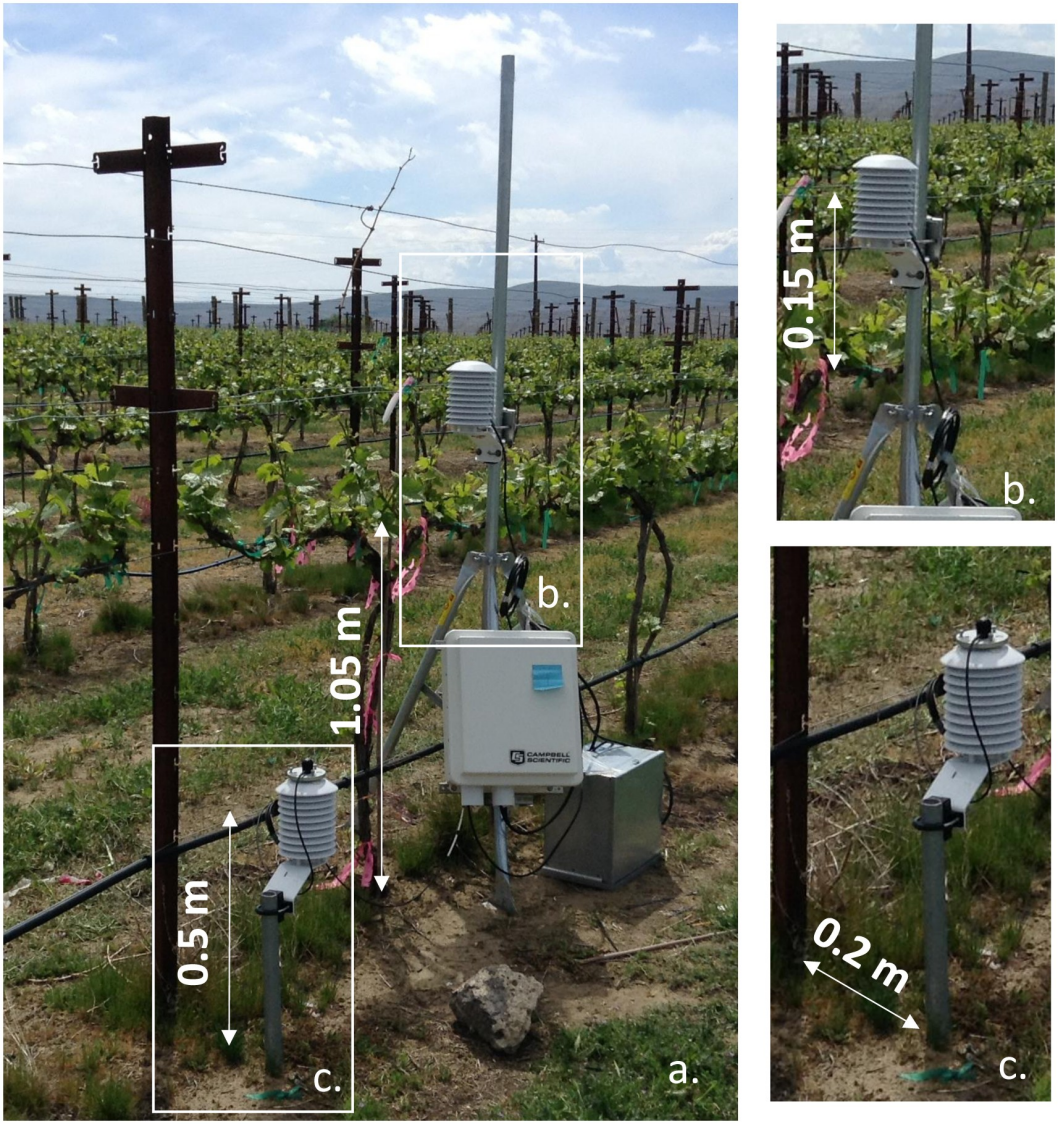
Temperature measurements within the vineyard (a). Temperature probes were installed at a height of 1.2 m above the soil surface and 0.15 m above the cordon (b) and at a height of 0.5 m above the soil surface and separated 0.2 m from the vine rows (c).

Seven precision thermistors enclosed in a waterproof rubber covering manufactured by Apogee (ST-100 Model, Logan, Utah, USA) were used for measuring canopy temperature. These thermistors are designed to measure temperature with a high degree of accuracy from -25 to +70°C. The sensors were installed on December 10, 2015: four at a height of 0.5 m and three at a height of 1.2 m above the soil surface. Vines in a commercial vineyard grow most of the canopy and produce most of the fruits between those heights (0.5 and 1.2 m). The measurements were terminated on June 30, 2017 (Fig 1). The thermistors were installed by using the same radiation shield that was employed at the automated weather station. The upper thermistors were installed above the cordon so that they were surrounded by leaves during the summer. The lower thermistors were installed 0.2 m from the trunks line (row) facing the east and west side of the rows, two on each side at a distance of 0.2 m from the trunk (Fig 1).

#### c. Measurements within the berry clusters

**Fig 2:**
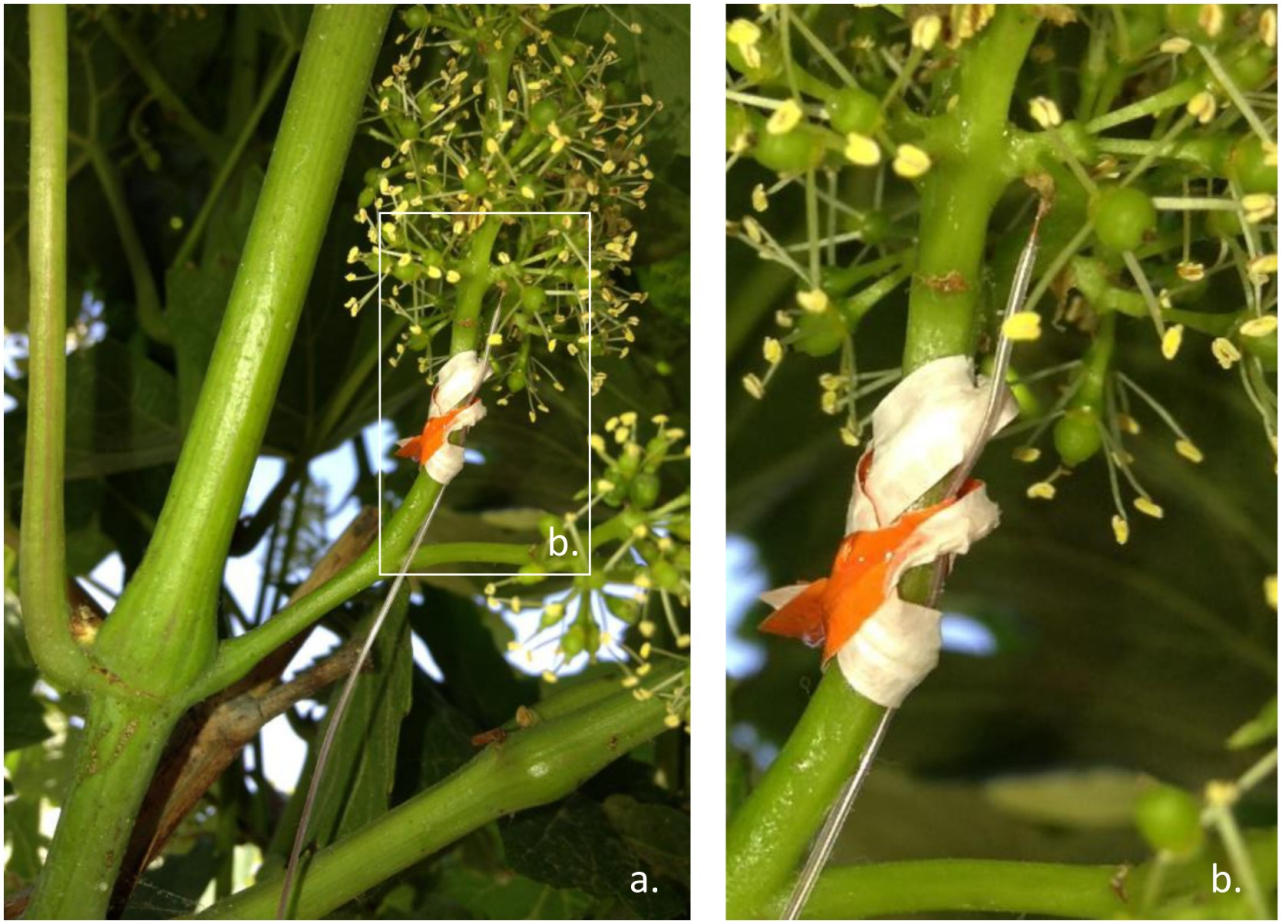
Probe installed to measure air temperature within berry clusters (a). “T type” thermocouples were installed in the rachis, close to the first or second branching point (b).

The air temperature within the clusters was measured with six precision type “T” thermocouples that had a diameter of 0.127 mm (gauge number 36) manufactured by Omega (Norwalk, Connecticut, USA). During 2016, measurements were taken for 106 days starting on June 1, between full bloom and harvest, and during 2017 measurements waken for 81 days starting on July 6, between the pea size phenological stage and harvest. The thermocouples were installed near the rachis and close to the first or second branching point to ensure that the sensor or junction of the thermocouple did not touch the berries (Fig 2). Three thermocouples were installed within berry clusters located on the east side of the canopy and the other three were installed on the west side of the canopy.

#### d. Data standardization

At the beginning of the experiment it was determined that the air temperature recorded by the thermocouples should be calibrated against the thermistors with a correction to subsequent data. Three thermocouples and two thermistors were placed in a 1007C Temperature Chamber (TestEquity, Moorpark, California, USA) and exposed to different temperatures. Thermistors were connected to a CR 1000, while thermocouples were connected to two CR10. Two CR10TCR devices (Campbell Scientific, Logan, Utah, USA) were connected to CR10 dataloggers, for junction reference. The chamber was programmed to simulate various daily air temperature profiles during four consecutive days characterized by four minimum/maximum temperatures: 12/38°C, 4/28°C, 0/16°C, and -10/6°C. The chamber started the simulations at 12°C, the minimum temperature of day one, and the heat increased linearly over a period of 12 hours until it reached 38°C, the maximum temperature of the first day. At that point, the temperature decreased linearly for 12 hours until it reached 4°C, the minimum temperature for the second day. This pattern continued until the maximum temperature of the fourth day was reached and maintained for 12 more hours. The temperature was recorded at 1-minute intervals and summarized every 15 minutes for 96 hours for a total of 384 observations (four days). The data were adjusted as described, and the 15-min data observed with the thermistors and thermocouples were compared (n = 384) (Eq 1).

$$\begin{array}{cc} TM_{15}=\left(0.9982\left(TC_{15}\right)\right)+0.4527 & {\text{R}}^{2}=0.9781 \end{array}$$ (1)

where *TM*_*15*_ is air temperature measured by the thermistor and *TC*_*15*_ is air temperature measured by the thermocouple.

#### e. Data recording

All air temperature probes used in this study, including those at the weather station, were connected to Campbell Scientific data loggers (Campbell Scientific, Logan, Utah, USA) to record the temperature over time. A CR-1000 device was used at the weather station, and five CR-10s were used for the canopy and berry measurements. Both types of data loggers were programmed to record each measurement at 5-second intervals and to summarize the data every 15 minutes. One channel of two data loggers, where thermocouples were installed, was used to connect two CR10TCR devices (Campbell Scientific, Logan, Utah, USA), which we used for junction reference.

### 2.3 Statistical analysis

#### a. Data

Five time series were analyzed at both 15-min and daily scales. The data included the air temperature measured at the weather station (T_station_1.7_), the air temperature within the canopy at a height of 0.5 m (T_canopy_0.5_), and at 1.2 m (T_canopy_1.2_), and the temperature within the berry clusters facing west (T_berry_west_) and facing east (T_berry_east_). The average of the daily maximum and minimum temperature measured by the sensors installed for the same location (T_station_1.7_, T_canopy_0.5_, T_canopy_1.2_, T_berry_west_, and T_berry_east_) was used to describe the response for the daily air temperature. For the 15-min scale, the analysis considered the average temperature of the period, obtained from 180 measurements recorded at a 5-second interval.

#### b. Mean comparison

T-tests were performed to estimate the difference in mean daily values of maximum and minimum air temperature and the thermal amplitude measured at the weather station and measured in the canopy and in the berry clusters. The t-test compared the average of the two independent groups to determine whether the difference between the mean values of T_station_1.7_ and the mean values of T_canopy_0.5_, T_canopy_1.2_, T_berry_east_ and T_berry_west_, were significant. The comparison was conducted for the entire series (n = 478 days). A second comparison was conducted between the maximum and minimum air temperature measured at the weather station and the maximum and minimum temperature measured in the canopy for the different seasons. For both comparisons, the null hypothesis was that the average of the values would be equal (μ_1_ = μ_2_) and the significance was set to p = 0.05.

#### c. Linear regression analysis

Linear regressions were conducted to compare the daily air temperature data measured in the canopy and the berry clusters with the daily air temperature measured at the weather station for both the daily and 15-min data. At the daily scale, the models were adjusted using the T_station_1.7_ as the independent variable (*x*) and T_canopy_0.5_, T_canopy_1.2_, T_berry_east_ and T_berry_west_, as the dependent variables (*y*). Scatter plots and linear modelling based on the maximum and minimum daily air temperature were performed by using the data collected between December 2015 and June 2017. The data at the 15-min scale were analyzed by adjusting linear models with T_station_1.7_ as the independent variable and T_canopy_0.5_ and T_canopy_1.2_ as the dependent variables. The linear regression performed with the15-min data was based on lags, known as cross correlation, which measures how canopy air temperature data relate to the air temperature data measured at the weather station as a function of the displacement of one series relative to the other. This type of analysis is commonly used in signal processing [28] where the peaks of the correlation reflect the correspondence between the time when the maximum or minimum air temperature occurred within the canopy and at the weather station. Nine lags were evaluated, ranging from -60 to +60 minutes, including a non-lag or lag 0. The predominance of a negative lag stands for the occurrence of maximum (minimum) temperature within the canopy prior to the occurrence of the maximum (minimum) temperature at the weather station.

## 3. Results

### 3.1. Air temperature within the canopy

#### a. Comparison of daily maximum and minimum temperature

Minimum air temperature measured at two heights within the canopy was similar for the four seasons of the year. There was no significant difference between maximum air temperature measured at a height of 1.2 m and the air temperature at 0.5m during most of the year. However, during the summer the air temperature measured at a height of 1.2 m above the soil surface was 0.5°C higher than the maximum temperature measured at a height of 0.5 m (Table 2a).

**Fig 3:**
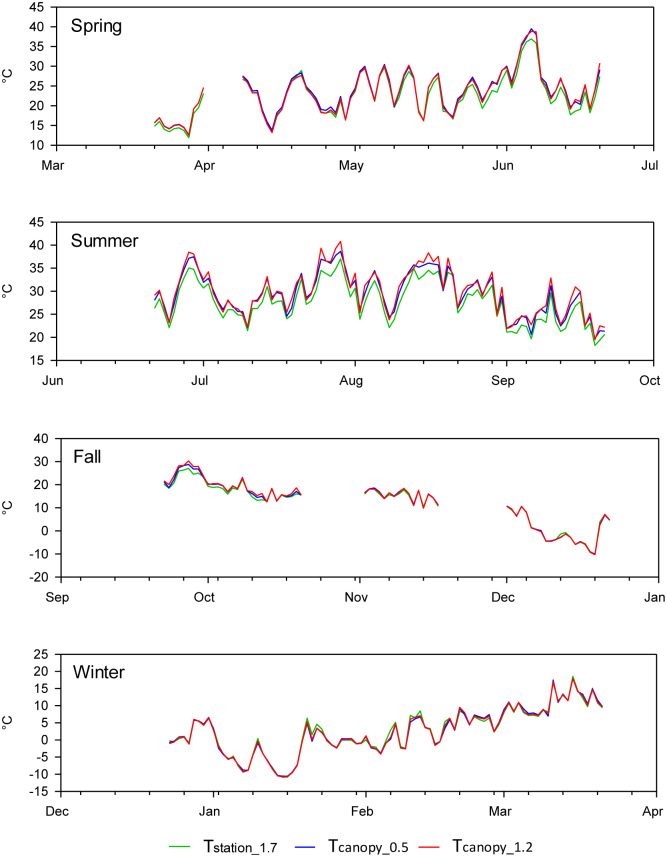
Time series of daily maximum air temperature measured between March 22, 2016, and March 21, 2017, at the weather station (T_station_1.7_), and within the vineyard canopy at a height of 0.5 m (T_canopy_0.5_) and 1.2 m (T_canopy_1.2_) above the ground surface.

**Fig 4:**
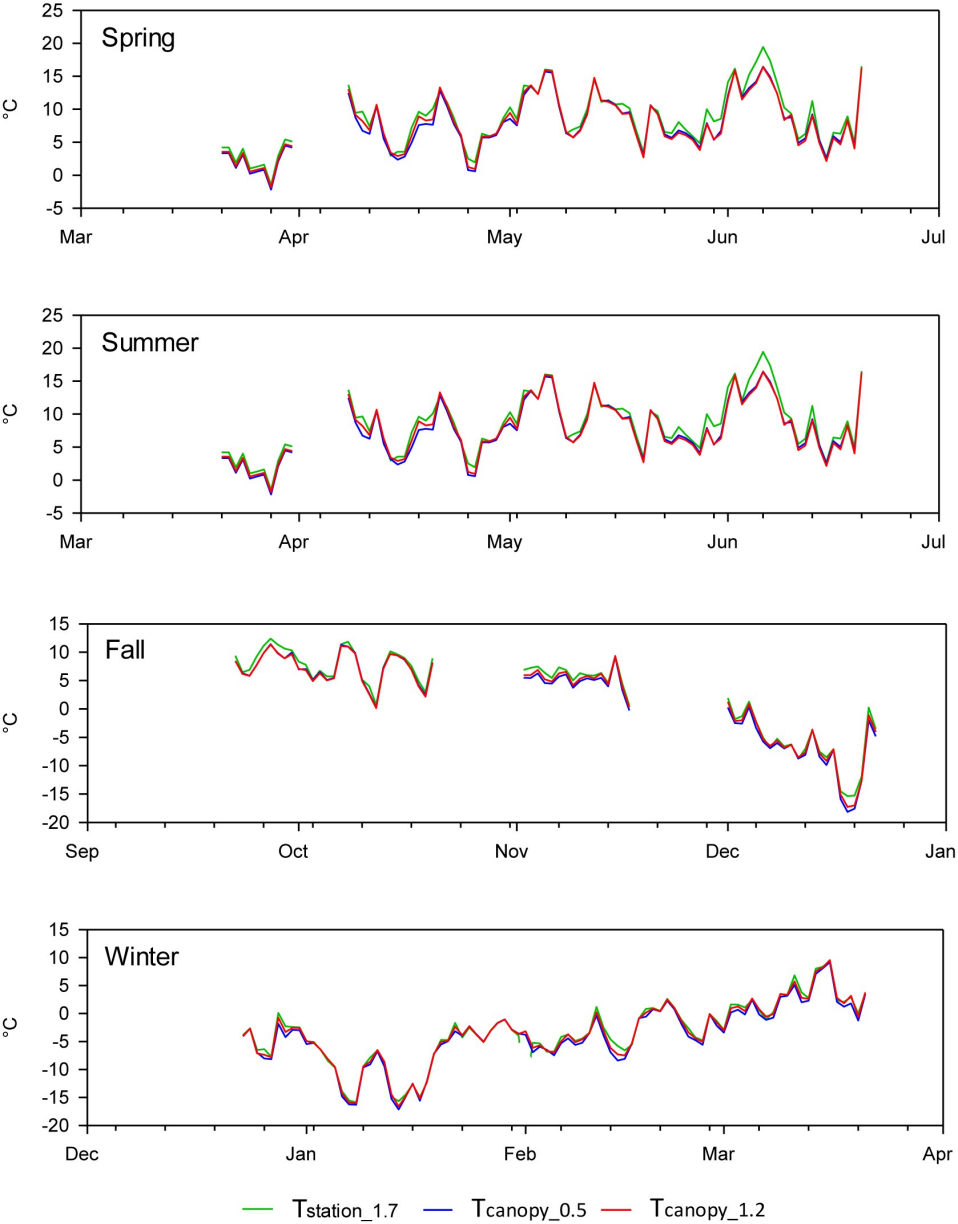
Time series of daily minimum air temperature measured between March 22, 2016, and March 21, 2017, at the weather station (T_station_1.7_), and within the vineyard canopy at a height of 0.5 m (T_canopy_0.5_) and 1.2 m (T_canopy_1.2_) above the ground surface.

The air temperature measured within the grapevine canopy differed from the air temperature recorded at the standard weather station. In general, the maximum temperature within the vine canopy was higher (Fig 3), while the minimum temperature was lower (Fig 4) compared to the air temperature recorded at the automated weather station. The differences between the maximum air temperatures were greater during the final days of spring, summer, and the first days of fall (Fig 3). Similarly, the differences between the minimum air temperatures were greater between the final days of spring and the first 15 days of fall (Fig 4). The analysis of the entire daily time series for the maximum and minimum air temperatures from December 2015 to June 2017, showed that there were no significant differences between the mean maximum or minimum air temperature measured within the grapevine canopy and measured at the standard weather station (Table 1). However, there was a significant difference between the mean values of the daily thermal amplitude, i.e., the difference between the maximum and minimum air temperature measured at the weather station and within the crop canopy. Therefore, we explored the differences and also considered the effect for each individual season. Only during the summer was there a significant difference between the mean minimum and maximum air temperature measured at the weather station and in the vineyard canopy (Table 2a). During the other seasons, the p-value showed that the difference between the air temperature measured in the canopy and at the weather station was not significant (p-values between 0.18 and 0.95). However, during spring, p-values were lower than during fall and winter (Table 2a), a pattern related to the differences between the series at the end of spring. The minimum air temperature in the canopy during the summer was generally 1.1 or 1.2°C lower than the minimum air temperature measured at the weather station. In contrast, during the summer the maximum air temperature measured in the canopy was 1.5 to 2°C higher than the maximum air temperature measured at the weather station (Table 2a).

**Table 1:** T-test comparison between daily air temperature measured under standard conditions (T_station_1.7_) and within the canopy at a height of 0.5 and 1.2 m above the soil surface (T_canopy_0.5_ and T_canopy_1.2_). The temperature was recorded between December 2015 and June 2017.

Variable	Difference		t-Value		p-Value	
	T_canopy_0.5_	T_canopy_1.2_	T_canopy_0.5_	T_canopy_1.2_	T_canopy_0.5_	T_canopy_1.2_
Minimum Temperature (°C)	-0.917	-0.744	-1.814	-1.489	0.075	0.137
Maximum Temperature (°C)	1.103	1.077	1.405	1.362	0.161	0.173
Thermal Amplitude (°C)	2.020	1.820	5.597	4.886	*0.000	*0.000

*Significance level

**Table 2:** T-test comparison between the daily air temperature measured under standard conditions (T_station_1.7_) and within the canopy. a. Comparison between T_station_1.7_ and air temperature within the canopy at a height of 0.5 (T_canopy_0.5_) and 1.2 m (T_canopy_1.2_) above the soil surface based on seasonal differences. The air temperature was recorded between December 2015 and June 2017. b. Comparison between the daily air temperature measured under standard conditions (T_station_1.7_) and among the berries in the clusters facing east (T_berry_east_) and west (T_berry_west_). The temperature was recorded between December 2015 and June 2017.

Season	Minimum Temperature (°C)					
	Difference	t-Value			p-Value	
	T_canopy_0.5_	T_canopy_1.2_	T_canopy_0.5_	T_canopy_1.2_	T_canopy_0.5_	T_canopy_1.2_
Spring	-0.885	-0.769	-1.339	-1.168	0.182	0.244
Summer	-1.140	-1.284	-2.692	-3.026	*0.008	*0.003
Fall	-0.868	-0.630	-0.684	-0.502	0.495	0.616
Winter	-0.481	-0.107	-0.584	-0.129	0.560	0.897
Season	Maximum Temperature (°C)					
	Difference	t-Value			p-Value	
	T_canopy_0.5_	T_canopy_1.2_	T_canopy_0.5_	T_canopy_1.2_	T_canopy_0.5_	T_canopy_1.2_
Spring	1.026	0.815	1.231	0.970	0.220	0.333
Summer	1.670	2.164	2.506	3.165	*0.013	*0.002
Fall	0.422	0.591	0.254	0.351	0.798	0.726
Winter	-0.066	-0.098	-0.067	-0.099	0.947	0.922
Variable	T_berry_east_					
	Difference	t-Value	p-Value			
Minimum Temperature (°C)	-1.43	-3.162	*0.002			
Maximum Temperature (°C)	1.674	2.567	*0.011			
Thermal Amplitude (°C)	3.104	6.529	*0.000			
Variable	T_berry_west_					
	Difference	t-Value	p-Value			
Minimum Temperature (°C)	-1.655	-3.665	*0.000			
Maximum Temperature (°C)	6.139	7.407	*0.000			
Thermal Amplitude (°C)	7.794	11.701	*0.000			

*Significance level

#### b. Linear regression analysis

**Fig 5:**
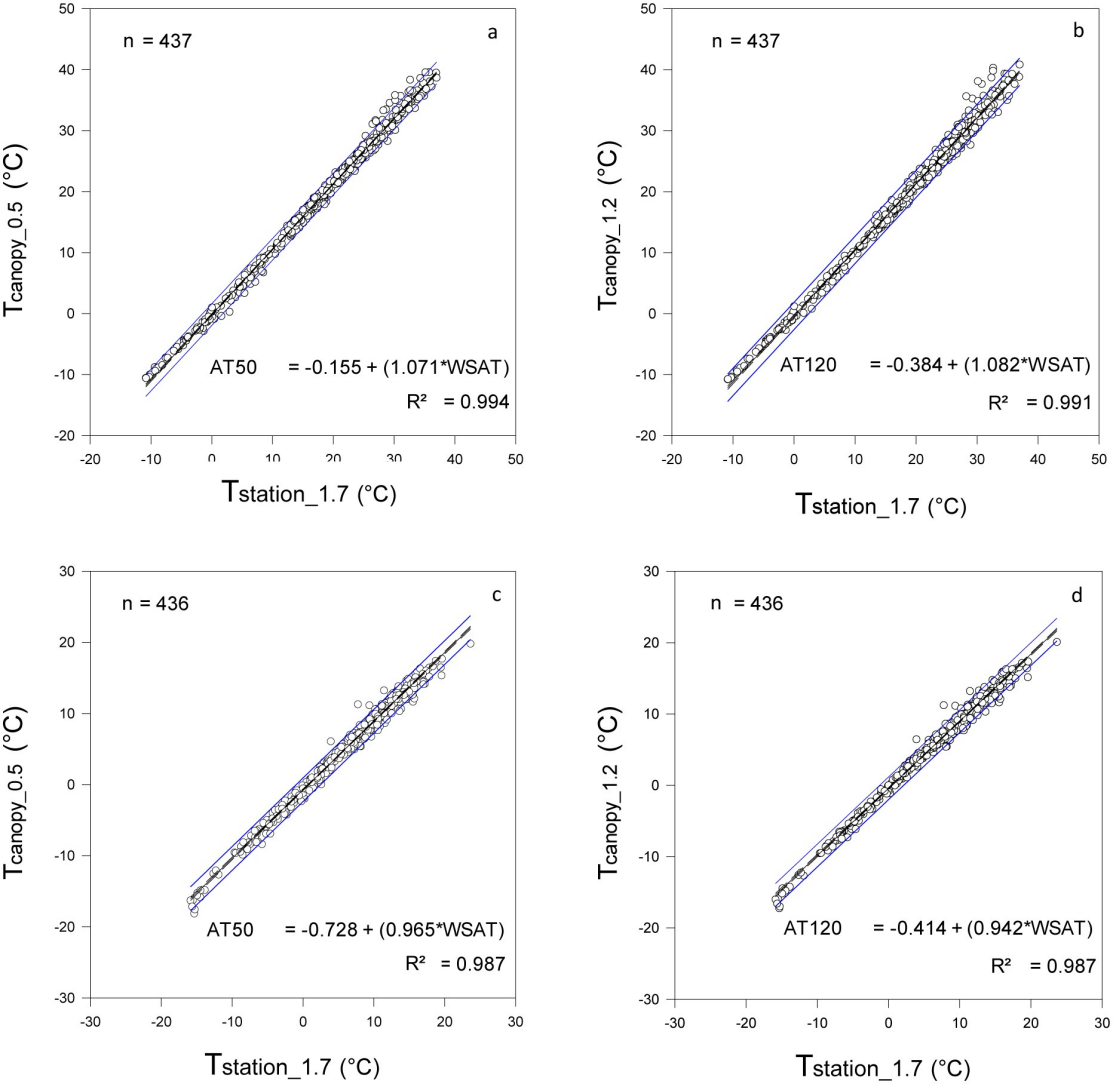
Linear regression between daily maximum (a and b) and minimum (c and d) air temperature measured at the weather station (T_station_1.7_) and within the canopy of the vineyard at a height of 0.5 m (T_canopy_0.5_) and 1.2 m (T_canopy_1.2_) above the soil surface.

An analysis of the daily maximum and minimum air temperature confirmed the seasonal response of the difference between those temperatures. The regression equation that was developed showed that the minimum air temperature of the canopy tended to be lower than the minimum air temperature measured at the weather station. Because the slope was less than one and the intercept was negative and significantly different from zero (p<0.06), the difference between the variables was consistent for the entire year. The air temperature in the canopy was lower than the air temperature measured at the weather station. However, the difference between T_station_1.7_ and T_canopy_ was higher and significant (p<0.05) during those days when the minimum air temperature measured at the weather station had the highest values. For example, during the summer the difference between T_station_1.7_ and T_canopy_ was around 1.5°C, but during the winter this difference tended to be zero (Fig 5c and 5d). The regression equation for the relationship between the maximum air temperature of T_station_1.7_ and T_canopies_ had an intercept that was not significantly different from zero (p<0.27), but a slope that was positive and significantly different from one (p<0.05), thus showing that T_station_1.7_ and T_canopies_ had different responses.

The maximum air temperature measured in the canopy was similar to the maximum air temperature measured at the weather station when T_station_1.7_ reached the lowest values during the winter. However, during other seasons, the maximum temperature within the canopy was higher than the maximum air temperatures measured at the weather station. This difference between T_station_1.7_ and T_canopies_ was higher when T_station_1.7_ reached values above 30°C. For example, during a summer day when the maximum air temperature measured at the weather station reached 35°C, the air temperature inside the canopy was close to 37.5°C, more than 2°C higher than the maximum air temperature measured at the weather station (Fig 5a and 5b).

**Fig 6:**
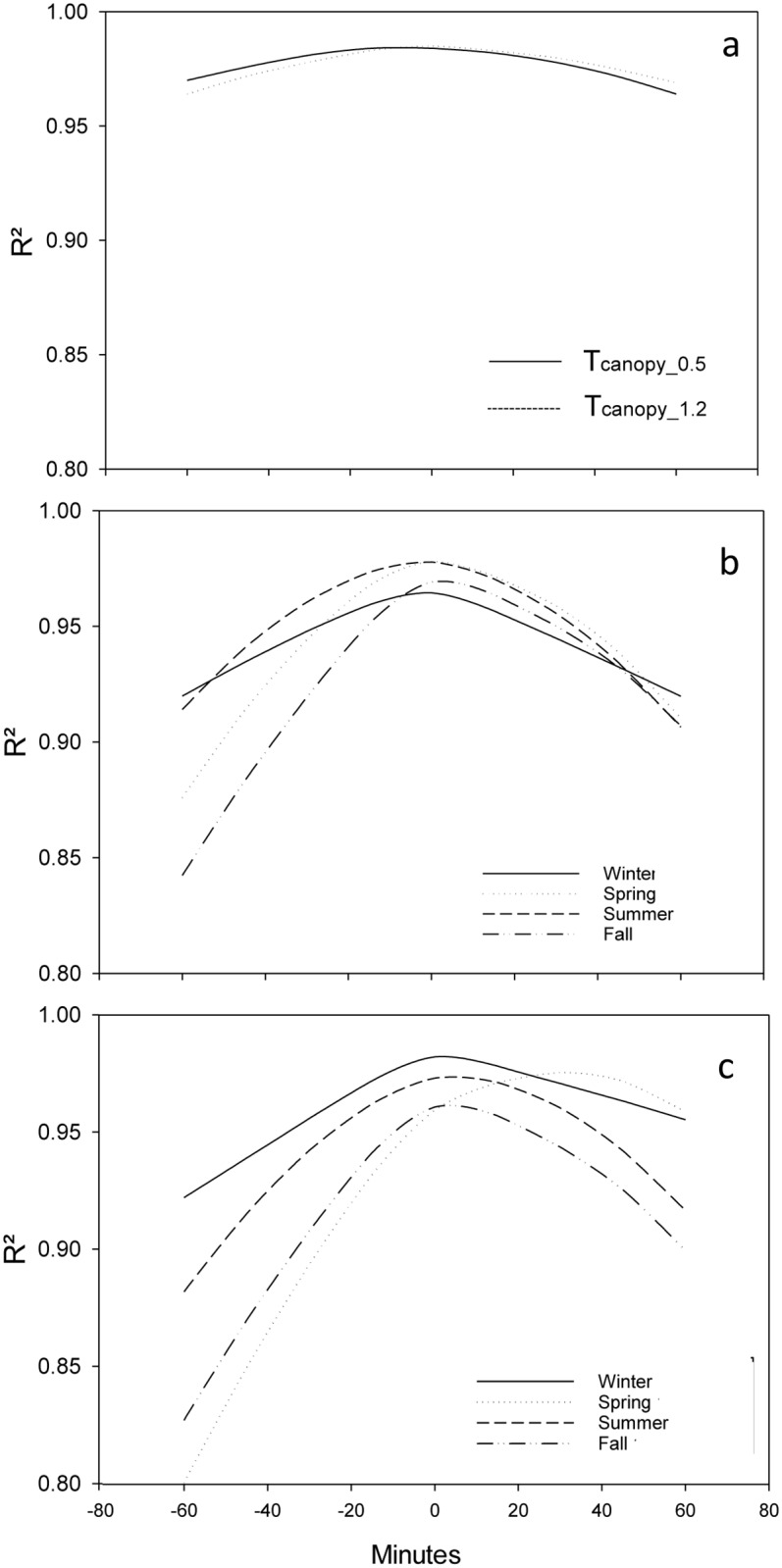
Cross correlation between the air temperature measured at two heights in the canopy and the weather station (a), and for the air temperature measured at 0.5 m (b) and 1.2 m (c) by season for the period December 2015 and July 2017. R^2^ is the coefficient of determination.

The linear regression for the 15-minute data showed that during the year, both the daily minimum and daily maximum air temperatures in the canopy and the weather station occurred simultaneously with no lag (Fig 6a). During the summer and winter, both the maximum and minimum air temperature had the same response as the general conditions mentioned previously, and the correlation peak was reached for lag 0, which means that there was a simultaneous occurrence of extreme temperatures in the canopy and at the weather station (Fig 6b and 6c). During fall, the relation between the timing of the maximum and minimum canopy air temperature and the maximum and minimum air temperature at the weather station was similar to the relations found for spring, with higher correlations with positive lags. This trend means that during the fall and spring, the maximum and minimum air temperature inside the canopy occurred after the maximum and minimum air temperature were observed at the weather station (Fig 6b and 6c). In fact, during spring the maximum and minimum air temperature were recorded between 30 and 45 minutes after the extreme values were recorded at the weather station (Fig 6c).

### 3.2. Air temperature within the berry cluster

#### a. Comparison of daily maximum and minimum temperatures

**Fig 7:**
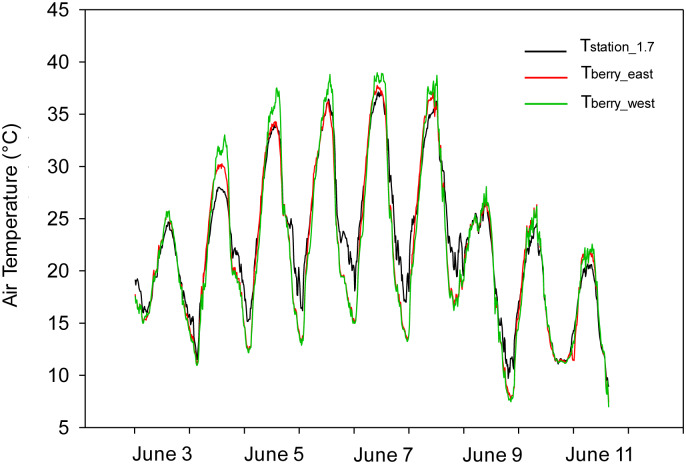
Example time series for 15-min air temperature measured at the weather station (T_station_1.7_) and the grape berry clusters facing east (T_berry_east_) and west (T_berry_west_) during 2017.

The differences between the air temperature surrounding the berry clusters and the air temperature measured at the weather station showed patterns that were similar to the daily differences between the canopy air temperature and the air temperature measured under standard conditions. During the day, the air temperature inside the cluster tended to be higher than at the weather station, making the daily maximum air temperature in the cluster higher than the maximum air temperature measured at the weather station (Table 2b). During the night, the air surrounding the clusters was cooler than the air temperature at the weather station. Therefore, the minimum air temperature measured in the clusters was lower than the minimum air temperature measured at the weather station (Table 2b). The clusters located on the west side of the canopy experienced higher temperatures during the day than those on the east side of the canopy, meaning that the maximum air temperature measured in the clusters facing west was higher than the air temperature measured in the clusters facing east (Fig 7). The minimum air temperature measured within the clusters was much lower than the minimum temperature measured at the weather station. The location of the berry cluster had no effect on the mean value of the difference between the minimum temperature of the cluster and the weather station which was around 1.5°C (Table 2b).

#### b. Linear regression analysis

**Fig 8:**
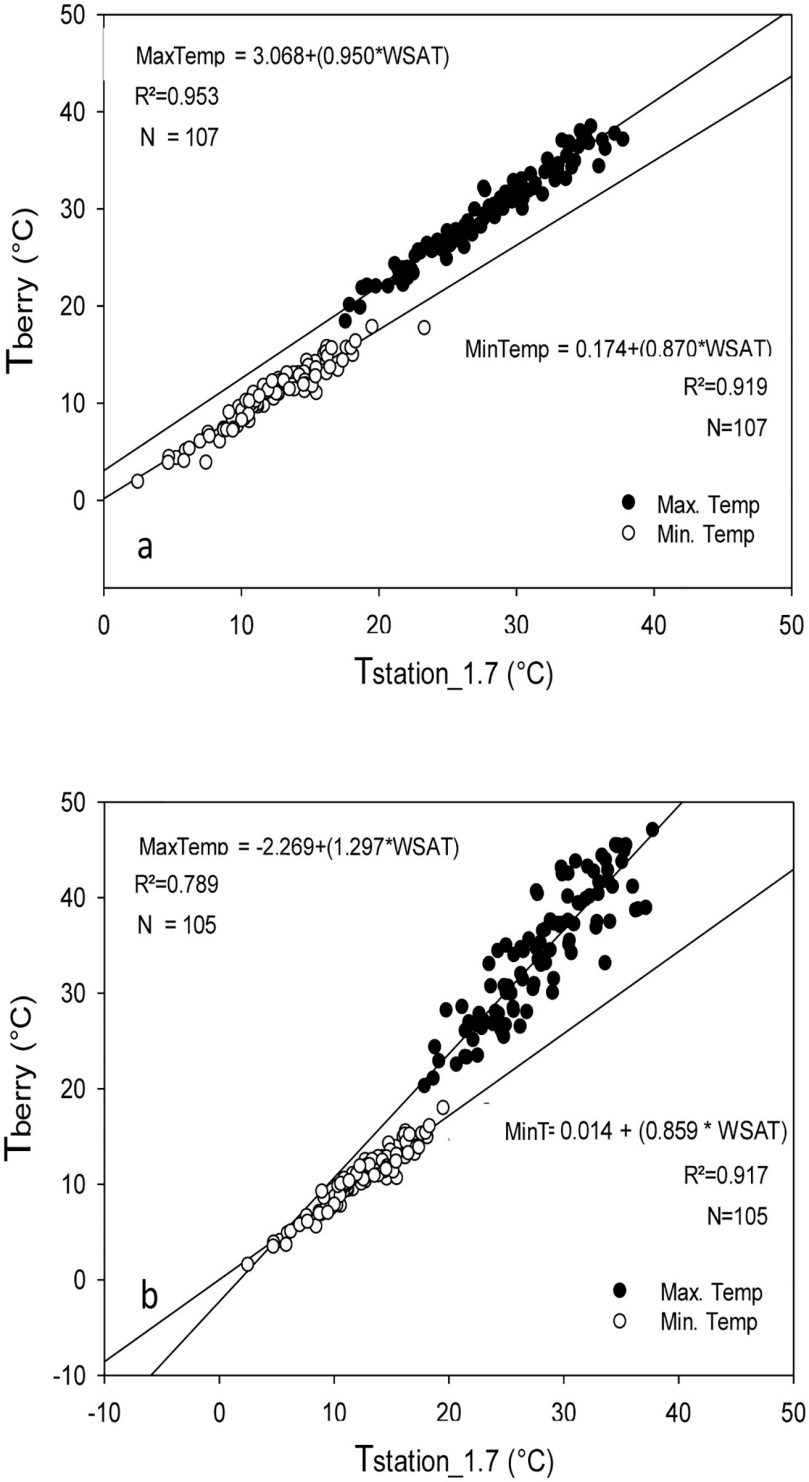
Linear regression comparing the daily maximum and minimum temperature measured within clusters (T_berry_) facing east (a) and west (b) in the vineyard and at the weather station (T_station_1.7_).

The intercept and slope of the linear equations that relate daily minimum air temperature measured within the clusters facing west and east and the minimum air temperature measured at the weather station showed that the minimum air temperature at the two sides was always lower than the one measured at the weather station. Moreover, there was not a significant difference between the parameters of both equations, and, therefore, no difference between the minimum air temperature values measured in clusters facing east and west (Fig 8). The parameters of the linear equations that describe the relation between the maximum air temperature measured within the clusters facing west and east and the maximum air temperature measured at the weather station were significantly different from those found for minimum temperature. In contrast to the minimum air temperature, the maximum air temperature within the berry clusters was higher than the air temperature measured at the weather station. However, the equations that relate T_station_1.7_ with T_berry_east_ and T_berry_west_ were different (Fig 8). For example, the maximum air temperature in the clusters facing east was linearly related to the air temperature at the weather station with a slope that was lower than one (p<0.10) and an intercept that was significantly higher than zero (p<0.01). The values of these two parameters not only represent higher values of temperature than at the weather station but also higher differences between T_station_1.7_ and T_berry_east_ during days with lower air temperature (Fig 8). For example, when the maximum air temperature measured at the weather station was 25°C, the maximum air temperature of the clusters facing east was about 26.7°C, or a 1.7°C difference. However, when the temperature recorded at the weather station was 35°C, the difference was only 1.2°C. The slope of the regression equation relating T_station_1.7_ and T_berry_west_ was higher than one (p<0.05), which was significantly higher than the slope that relates T_station_1.7_ and T_berry_east_ (Fig 8). Moreover, the intercept of the equation that relates T_station_1.7_ and T_berry_west_ was lower than zero (p<0.01). This configuration in the parameters of the regression equations means that the air temperature of the berries exposed to the west had greater differences during days with a high air temperature, especially during the summer (Fig 8).

**Fig 9:**
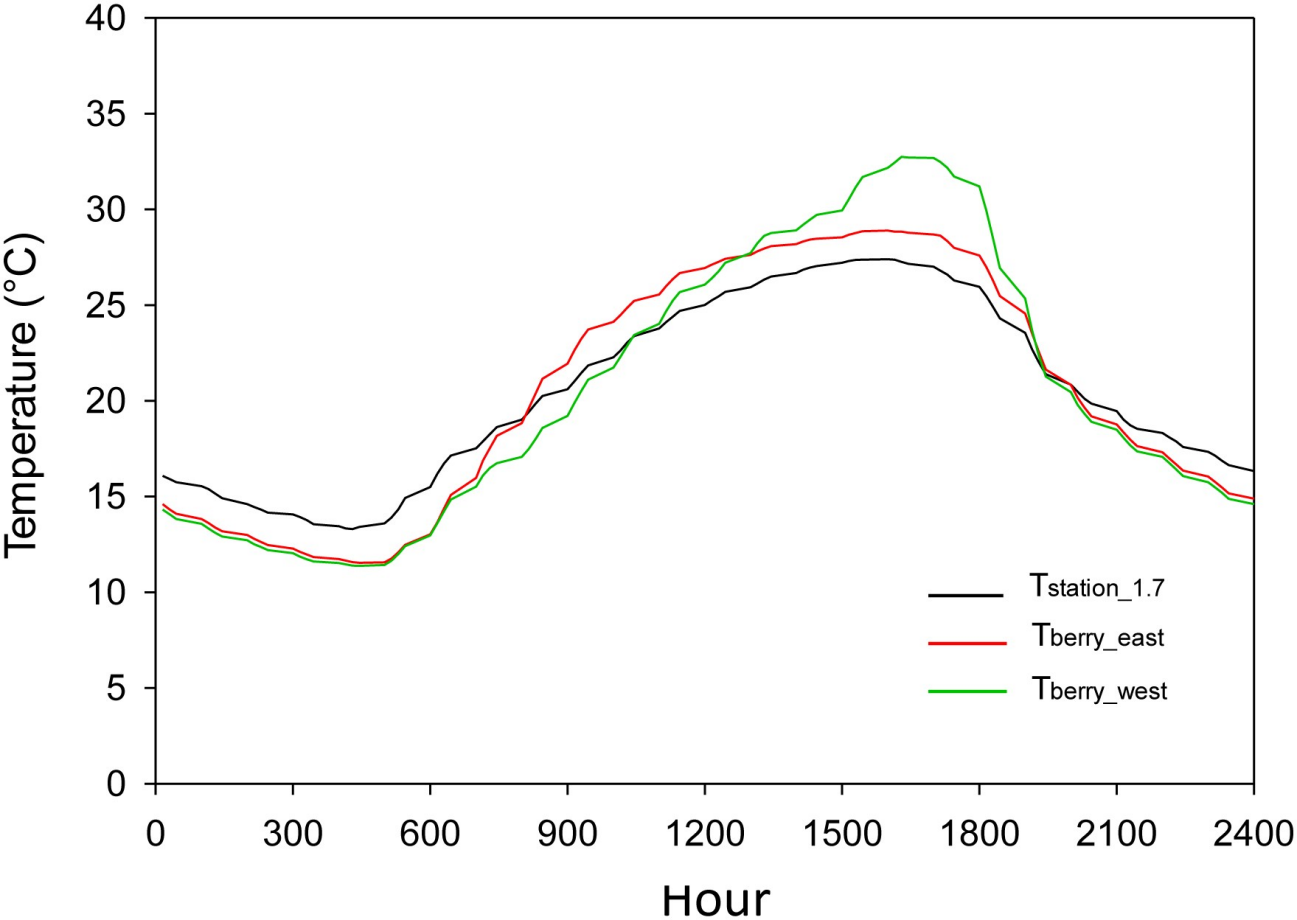
Typical daily profile of air temperature, based on 15-min data, of the ambient in the clusters facing east (T_berry_east_) and west (T_berry_west_) and at the weather station (T_station_1.7_).

The analysis at the 15-min scale showed that there was no difference between the air temperature measured within the clusters facing east and west during the early morning, similar to what was discussed previously. The air temperature within the clusters was lower than the air temperature measured at the weather station, a condition that starts to change two to three hours after sunrise in the east side clusters. After that time, the air temperature within the clusters facing east became higher than the air temperature measured at the weather station (Fig 9). However, the air temperature surrounding the berry clusters facing west continued to be colder than the air surrounding the sensors at the weather station for one to two more hours. Between noon and 1 pm, the air temperature in the clusters facing west became warmer than the air temperature in the clusters facing east. During a typical day, the air temperature surrounding the berry clusters facing west was higher than the air temperature measured at the weather station for about nine hours. Meanwhile, the air temperature measured in the clusters facing east was higher than the air temperature measured at the weather station for about 12 hours (Fig 9). After the maximum temperature was reached, the clusters started to cool down. The cooling rate was higher for the west than for the east side, and the air for both the east and west clusters became simultaneously colder than the air at the weather station one hour after sunset (Fig 9).

## 4. Discussion

As expected, the air temperature measured in the canopy of the grapevines tended to be different from the air temperature measured at a weather station and this difference was higher between late spring and early fall. The radiative heat transfer between the leaves and the air surrounding the canopy of a vineyard planted in a north-south direction accounts for a considerable proportion of the loss of energy from the canopy surface [29; 30]. As a result, during active canopy growth and expansion the maximum air temperature within the canopy is higher than the air temperature recorded at the weather station (Tables 1 and 2). During the winter when there are no leaves, the energy flux within the vineyard and the energy flux at the weather station are both dominated by the characteristics of the ground surface. Therefore, there were no differences between the minimum and maximum air temperature recorded in both environments (Table 2a). The increase in leaf area during late spring and summer increased the heat exchange between the leaves and the atmosphere, generating a greater difference between the air temperature measured at a height of 0.5 and 1.2 m in the canopy and defining the microclimate of the canopy during the grapevine growing season (Table 2a). These results concur with those reported by Sinoquet and Le Roux [14], who showed how the air temperature profile in tree plantations followed a thermal inversion. Transpiration also plays a significant role in the cooling of the leaves [8; 31; 29].

The daily minimum air temperature within the canopy was less variable throughout the different seasons than the maximum air temperature, while the minimum air temperature recorded inside the vineyard canopy was always lower than the minimum air temperature recorded at the weather station (Table 2a and Fig 5), which illustrates the classic definition of sensible heat flux [32; 33]. The impact of other atmospheric conditions such as wind, relative humidity, and the difference in height between the surface of the canopy, the sensors installed in the vineyard canopy, and the sensors installed at the weather station can mean a significant difference in sensible heat flux, generating differences between the canopy and the conditions at a standard weather station where there is free air movement. Assuming a linear relationship between air temperature and grapevine growth [34], the use of air temperature measured at the weather station as input for grapevine growth and development models could result in an underestimation of the real values for total leaf area and shoot length [35], which are traits that determine gross productivity for grapes.

The air temperature within the clusters was lower than the air temperature measured at the weather station during the morning, while during the day the air temperature in the clusters was higher than at the weather station (Table 2b and Fig 7). The daily maximum and minimum temperature of the air surrounding the berries in a cluster depend on the solar elevation. Because only 1 to 2% of the energy absorbed by the surface of the canopy is transformed into chemical energy [36; 29], the convective heat transfer as a process for dissipating excess energy is enhanced during the morning for the clusters facing east, causing an increase in the surrounding air temperature of the berries. There is, therefore, a sensible heat flux from the surface of the berries in the cluster to the air surrounding the cluster. The same process also explains the increase in the temperature of the air surrounding the berry clusters facing west as the sun changes its orientation during the day, with the west-facing berry clusters exposed to the direct sun in the afternoon (Figs 8 and 9). The air temperature surrounding the berries showed the same daily trend as the skin temperature of the berries, which is supported by studies conducted by Spayd et al. [12] and Van Zyl and Van Huyssteen [37], who found an increase in air temperature inside the vineyard during the afternoon. The different slopes of the regression models for maximum air temperature (Fig 8) can be explained by the heat transfer of the clusters directly exposed to sunlight, with the air pockets serving as the primary receptor of heat during the convection [24; 12]. Therefore, the air temperature surrounding the berries on the east side of the canopy was higher than the air temperature surrounding the berries on the west side of the canopy from sunrise to solar noon. However, from solar noon to sunset the temperature of the berries on the west side was higher than the air temperature of the berries on the east side. At night, the air temperature of the berry cluster was lower than the air temperature at the weather station because of the low capacity of the berries to keep the heat that had been gained during the day; this became apparently immediately following sunset (Fig 9). This contradicts the reported low thermal effusivity of plant leaves [38]. A low effusivity means that there is limited ability to exchange thermal energy, while materials with high thermal effusivity cannot hold heat long enough from the surface when the surrounding temperature drops [39]. The relation between the presence of extreme temperatures inside and outside the canopy that was found in this study (Fig 6) suggests that, at least for Chardonnay, the leaves cannot hold the absorbed heat and, thus, is rapidly released at night. This high rate of heat release could be enhanced by the wind velocity. Sinoquet and Le Roux [14] found that the air temperature around trees is the result of instantaneous effects of other variables affecting the trees, such as solar radiation, wind speed and direction, and the shape and size of the canopy. The wind speed at the Roza weather station tended to be higher between 10 pm and 1 am from July and until September, thus enhancing the vertical turbulent flux.

The differences between the temperature of the air surrounding the clusters facing east and west during the days prior to Veraison could determine different estimations of the chemical composition of grape berries, especially pH and soluble solids. The difference between east and west can also determine alterations in the mass of the berries, which are part of the clusters facing west due to the higher number of hours exceeding 30°C between bunch closure (July 21, 2016, and July 15, 2017) and harvest [12].

The linear models that relate maximum and minimum air temperatures within a vineyard canopy with maximum and minimum air temperatures at the weather stations showed high (R>0.90) correlations with no lag time (Fig 6). The simultaneous occurrence of maximum and minimum air temperatures within the canopy and at the weather station suggested that the processes that explain the variability of the air temperature within the canopy and at the weather station are the same. Therefore, the use of equations for estimating onsite air temperature based on air temperature data of the weather station should be a good option in the future. Knowing that the processes that lead to a variation in air temperature within the canopy and at the weather station are similar, the slope represents the difference between the response of the grapevine canopy surface to the factors affecting the temperature variation in comparison to the factors affecting the variation of temperature at the weather station. The intercept is the parameter that represents the consistency of the difference between the response in the canopy and at the weather station. For example, with a slope that is less than one and an intercept that is less than zero (Fig 5), the difference between the values of the minimum temperature measured in the canopy and at the weather station was consistent during the year. This means that most of the time the minimum air temperature in the canopy is lower than the minimum air temperature measured at the weather station. In contrast, the intercept of the equation obtained to estimate values of maximum air temperature in the canopy using data from the weather station was not significantly different from zero, and it was also negative (Fig 5). Therefore, the differences between the maximum air temperature in the canopy and the maximum air temperature measured at the weather station depend on the air temperature measured at the weather station. Hence, the difference is higher during days with high air temperature, but the difference will be lower during periods with lower air temperature. Thus, the maximum air temperature estimated using the equations will be higher than the maximum air temperature measured at the weather station during the summer, but during the winter the difference between the temperatures will be smaller.

In general, the results of this study show that there is a difference between the temperature of the air surrounding the vines and the air temperature measured at weather station. However, in the future it might be possible to determine the on-site air temperature based on the observations of a nearby weather station by using models that only consider air temperature measured at the weather station as input. Based on the results from this study, canopy management of grapevines and row orientation can affect air temperature within the canopy [40] and should be integrated into a strategy for improving productivity and quality of grape vines.

## 5. Conclusions

The daily maximum and minimum air temperatures measured within the grapevine canopy were different from the daily maximum and minimum air temperatures measured at a standard automated weather station installed close to the vineyard; these differences were significant (p<0.05) between late spring and early fall. The air temperature measured in the berry clusters was different from the air temperature measured at the weather station, and the difference was greater for the clusters facing west. These findings show that models assuming that air temperature measured at a weather station is not different from air temperature measured in the environment surrounding the vineyard could have greater uncertainty than models considering on-site air temperature data. While modelers try to improve the models and climatologists try to improve the quality of data for models, an issue associated with the users' assumption could be underlying model errors and uncertainty.

## Supporting information

S1 File (XLSX)
